# Distinctive Roles of Medial Prefrontal Cortex Subregions in Strategic Conformity to Social Hierarchy

**DOI:** 10.1523/JNEUROSCI.0549-23.2023

**Published:** 2023-09-06

**Authors:** Daeeun Kim, JuYoung Kim, Hackjin Kim

**Affiliations:** ^1^Laboratory of Social and Decision Neuroscience, School of Psychology, Korea University, Seoul 02841, Republic of Korea; ^2^Department of Psychology, Korea Army Academy at Yeongcheon, Yeongcheon 38900, Gyeongsangbuk-do, Republic of Korea

**Keywords:** allostasis, decision-making, fMRI, observation, mPFC

## Abstract

People often align their behaviors and decisions with others' expectations, especially those of higher social positions, when they are being observed. However, little attention has been paid to the neural mechanisms underlying increased conformity to the social hierarchy under social observation. Using a preference rating task, we investigated whether and how individual preferences for novel stimuli were influenced by others' preferences by manipulating others' social hierarchy and observational context. The behavioral results showed that human participants of both sexes were more likely to change their preferences to match those of a superior partner in a public than in a private context. fMRI data revealed distinct contributions of the subregions of the medial prefrontal cortex (mPFC) to increased conformity to social hierarchy under observation. Specifically, the ventral mPFC showed increased activity when participants' preferences aligned with those of superior partners, regardless of behavioral manifestation. The rostral mPFC showed increased activity when conforming to a superior partner and nonconforming to an inferior one, indicating goal-dependent valuation. The dorsal mPFC showed increased activity in private conditions with a superior partner but only in those with a higher tendency to conform. These findings support the hierarchical allostatic regulation model of the mPFC function for social valuation and suggest strategic conformity as a way to minimize metabolic costs.

**SIGNIFICANCE STATEMENT** This study revealed distinct roles of subregions of the mPFC in increased conformity to individuals of different social ranks under observation. Specifically, the ventral mPFC showed increased activity when participants' preferences aligned with those of higher-ranking partners, whereas the rostral mPFC showed increased activity when conforming to a superior partner and nonconforming to an inferior partner, indicating goal-dependent valuation. The dorsal mPFC was more active in private conditions with a superior partner but only in those with a higher tendency to conform. These findings support the hierarchical allostatic regulation model of the mPFC function for social valuation and suggest strategic conformity as a way to minimize metabolic costs.

## Introduction

Social hierarchy is a ubiquitous principle in human societies, and most people belong to one or more groups that have a social hierarchy (Sapolsky, 2005; Qu et al., 2017). Because social hierarchy can profoundly affect the quality of life (Sapolsky, 2005), people often pay attention to the social hierarchy in the group they belong to for successful adaptation, improved personal or group performance, and positive relationships (French and Raven, 2015; Copeland, 1994; Chiao et al., 2004; Koski et al., 2015). Supporting this idea, it has been shown that human decision-making in a hierarchical context is influenced by the opinions, preferences, and attitudes of others in higher social positions, which is often called social conformity (Deutsch and Gerard, 1955; Cialdini and Goldstein, 2004; Galinsky et al., 2008; Hays and Goldstein, 2015; Qi et al., 2018; Li et al., 2021).

Why might psychological motivation underlie social conformity with others of higher social ranks? Perhaps people are motivated toward greater accuracy by placing more confidence in the opinions or decisions of those who rank higher in the social hierarchy, which is linked to a social phenomenon known as captainitis (Foushee, 1984; Cialdini and Goldstein, 2004). In addition, people may wish to make a better impression on those with higher social status by conforming to their opinions. For example, people were more inclined to change their preferences to align with those of superiors than of inferiors, especially when their decisions were visible to the superior but not the inferior others (Kim et al., 2021), suggesting that social conformity to those who rank higher in the social hierarchy may be mainly driven by a strategic motivation to make better impressions.

Conformity can be viewed as a type of reward-seeking behavior (Ruff and Fehr, 2014), given that social approval by others can reinforce conforming behavior (Baumeister and Leary, 1995; Cialdini and Goldstein, 2004; Klucharev et al., 2009). Supporting this idea, increased activity in the ventromedial prefrontal cortex (vmPFC) and the ventral striatum (VS), two key neural structures associated with reward processing, (Bartra et al., 2013) has been implicated in updating one's preferences to align with those of others (Klucharev et al., 2009; Campbell-Meiklejohn et al., 2010; Zaki et al., 2011; Chung et al., 2015; Nook and Zaki, 2015). Therefore, agreement with others or group opinions may be a strong driver of the reward system of the brain.

Unlike the vmPFC, the dorsomedial prefrontal cortex (dmPFC; Klucharev et al., 2009; Zaki et al., 2011; Izuma and Adolphs, 2013; Chung et al., 2015; Wu et al., 2016), in conjunction with the temporoparietal junction (TPJ; Campbell-Meiklejohn et al., 2010; Kang et al., 2013), is related to deviations from social norms and reactions to disagreements. For example, people who were more sensitive to social influence showed greater activity in the dmPFC and TPJ when they faced disagreements with experts or superior partners (Campbell-Meiklejohn et al., 2010). Additionally, functional connectivity between these two regions has been associated with higher accuracy in estimating others' preferences (Kang et al., 2013). The neural circuitry centered on the dmPFC and TPJ plays a crucial role in external valuation (Kim, 2020), and the increased activity in this circuitry when choosing to differ from others likely reflects an increased need to rely on additional external information.

The rostromedial prefrontal cortex (rmPFC), which is found between the vmPFC and the dmPFC, has been reported to track, learn, and update moment-by-moment knowledge about social hierarchy (Kumaran et al., 2016; Ligneul et al., 2016; Qu et al., 2017). It is not surprising that people are particularly attentive to the relative differences in the hierarchy between themselves and others as social hierarchy highly affects individuals' social and physical welfare within the group (Sapolsky, 2005). For example, people not only track and update their hierarchy (Zink et al., 2008; Kumaran et al., 2016; Li et al., 2021) but also pay attention to the social hierarchies of others to achieve successful outcomes (Ligneul et al., 2016). Furthermore, the rmPFC is particularly sensitive to social observations, such as public versus private conditions (Izuma et al., 2010; Müller-Pinzler et al., 2015; Hoorn et al., 2016; Jung et al., 2018; Yoon et al., 2021), which can increase the motivation to follow social norms (Deutsch and Gerard, 1955; Cialdini and Trost, 1998; Ariely et al., 2009; Bereczkei et al., 2010; Case et al., 2018; Lee et al., 2018). Several neuroimaging studies have demonstrated that the rmPFC is engaged when people are observed by others, leading to more prosocial behavior (Bereczkei et al., 2010; Izuma, 2012; Jung et al., 2018) or enhanced impression management (Izuma et al., 2010; Yoon et al., 2021). In other words, people are more inclined to employ socially acceptable strategies to achieve a positive impression or reputation in the presence of others (Bereczkei et al., 2010; Izuma et al., 2010; Izuma, 2012; Jung et al., 2018; Yoon et al., 2021).

These findings agree well with our previous proposal that the mPFC subregions are differentially involved in social valuation (Kim, 2020). In this model, the mPFC subregions interact with each other in such a way that more dorsal regions (i.e., the dmPFC) use additional external sensory information from the environment to predict and prevent conflicts occurring in the ventral regions (i.e., the vmPFC) tuned to internal bodily signals, thereby exerting hierarchically organized allostatic regulatory control over homeostatic reflexes. According to this model, the rmPFC plays a key role in adjusting internal (bodily) needs to better suit the constraints of external (environmental) variables.

In the present study, therefore, we primarily hypothesized that neural activity in the rmPFC would be engaged in strategically conforming behavior to a superior's opinions depending on the observational context. Additionally, we expected this neural signal to be associated with individual differences in strategic conformity, based on its role in shaping the internal drive for maintaining bodily homeostasis within the constraints of external or environmental contextual variables, as proposed by the hierarchical allostatic regulation model of the mPFC subregions for social valuation (Kim, 2020). To test our hypotheses, we provided participants with a social environment in which they had the opportunity to adjust their preferences to align with those of either superior or inferior partners under social observation, similar to our previous behavioral study (Kim et al., 2021), and examined their preference changes and neural activity using functional magnetic resonance imaging (fMRI).

## Materials and Methods

### Participants

Forty-seven participants were recruited via the Korea University community website for the fMRI experiment. Two participants were excluded from all analyses because they reported suspicion of the cover story. The final sample consisted of 45 participants (24 females; mean age, 22.5 ± 1.8 years). To determine the appropriate sample size for this study, we conducted an a priori power analysis with G∗Power version 3.1.9.6 software (Faul et al., 2007) based on the mean effect size of the interaction effects ($\eta_{p}^{2}$ = 0.17) that were drawn from the previous study on social conformity (Kim et al., 2021). The power analysis yielded that the required sample size at α = 0.05 with 95% power was *N* = 15. None of the participants had any history of neurologic, cognitive, or psychiatric disorders. No participant exhibited excessive head movements inside the MRI scanner with a threshold set at >3 mm. The experimental procedure was approved by the Institutional Review Board of Korea University, and all experiments were performed in accordance with the ethical guidelines and principles of the Declaration of Helsinki. Written informed consent was obtained from all participants before the behavioral experiment. All participants were compensated with 35,000 Korean won ($31.50).

### Experimental procedure

On arrival at the laboratory, the participants were provided with an overall description of the experiment. Before participating in the first hierarchy manipulation task, participants were informed that they would play an online game with two other participants located in separate rooms. The participants were given no information about the identities of the other participants. They were told that they would participate in three phases, (1) social hierarchy manipulation, (2) a preference rating task, and (3) a modified dictator game. All activities were programmed and run using MATLAB 2019b (MathWorks) and Cogent Graphics software packages. Before starting the social hierarchy manipulation and preference rating task, the participants were presented with a screen indicating that two other participants in separate laboratory rooms were accessing the online tasks to make the participants believe they were interacting with real human partners.

Time estimation and visual discrimination tasks were used to manipulate the participants' social hierarchy before the main task (Kim et al., 2021). Manipulating social hierarchy based on performance in a simple perceptual task has been shown to successfully engage participants in a manipulated social hierarchical context (Zink et al., 2008; Boksem et al., 2012; Santamaría-García et al., 2014). Participants were informed that their performance on these two perceptual tasks would determine their hierarchy in all subsequent experiments. However, in reality, all the participants were assigned an intermediate rank between their imaginary partners. To ensure the believability of the hierarchy manipulation, both tasks were designed so that it was difficult to assess one's real performance. Before the task, participants were informed that their final decisions in the public condition trials would be viewed by both superiorly and inferiorly ranked partners immediately on completion of the preference rating task. In contrast, their final decisions remain anonymous in private conditional trials.

**Figure 1.**
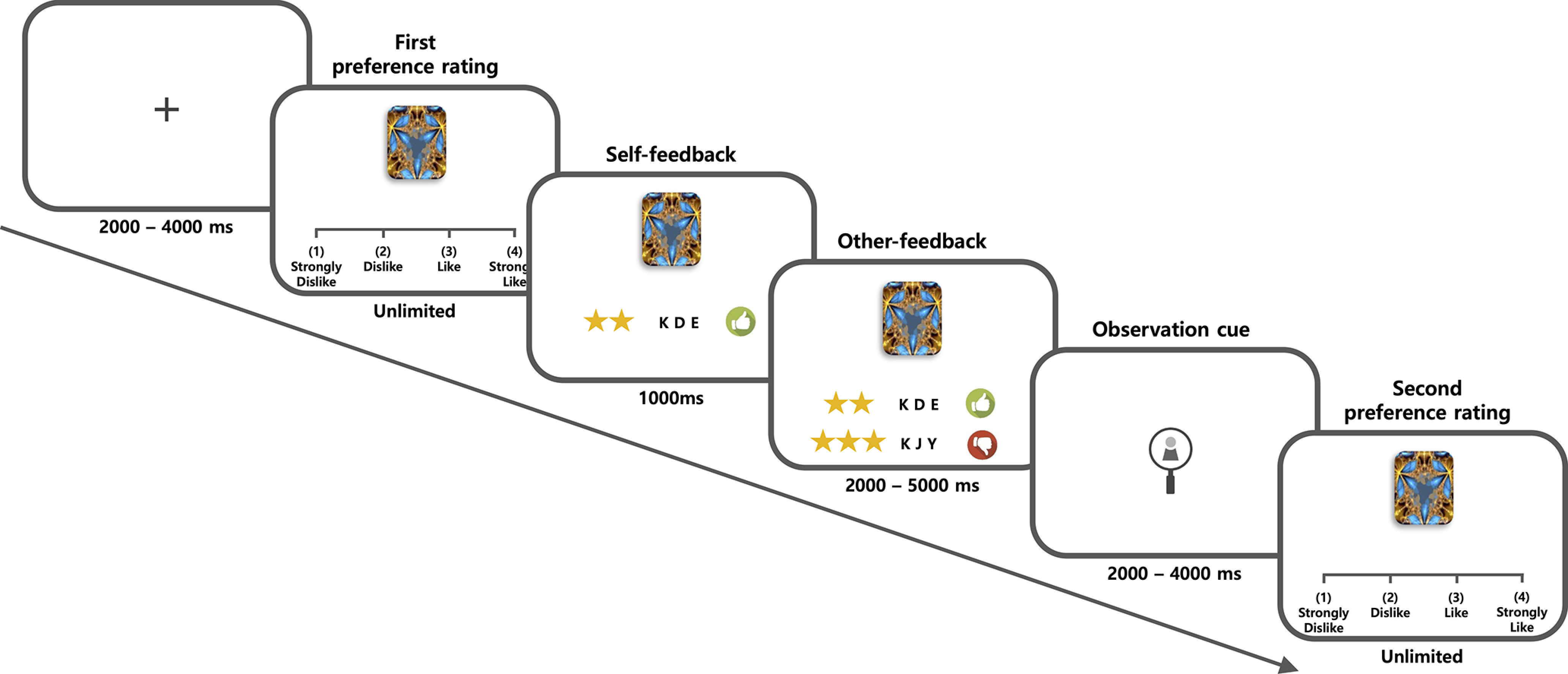
Schematic diagram of the preference rating task and behavioral results. Before the task, participants were informed that their final decisions in the public condition trials would be viewed by both their superiors and inferiors immediately after the end of the preference rating task and before the modified dictator game. In contrast, participants were informed that in the private condition trials, their preference ratings would remain completely anonymous. Each trial of the task consisted of five distinct phases.

Before entering the fMRI scanner, participants performed 10 practice trials. In the fMRI scanner, participants were presented with a log-on screen and asked to rate 120 fractal images for the main preference rating task. Each trial of the preference rating task (Fig. 1) consisted of five distinct phases. First, following the presentation of a jittered black fixation cross (2–4 s, with uniform distribution), a fractal image was presented on a 4-point Likert scale, and the participant was given an unlimited amount of time to evaluate and rate the likability or dislikability of the image (i.e., first preference rating). Second, the participants' ratings were displayed visually with either a thumbs-down image (rating of 1 or 2) or a thumbs-up image (rating of 3 or 4) for one second (i.e., self-feedback). Third, either the superior's or the inferior's preference rating of the same fractal image was displayed (2–5 s); the ratings were determined so that incongruent and congruent ratings relative to the participant's decision would be evenly distributed (i.e., other-feedback). Fourth, a symbol was shown to remind the participants that they were in either the public condition (an image of an eye in a magnifying glass) or the private condition (an image of a padlock, 2–4 s; i.e., observation cue). Finally, the same fractal image was shown again on a 4-point Likert scale, prompting the participant to evaluate and rate the image again with an unlimited amount of time permitted for responding (i.e., second preference rating).

The experimental design was organized into eight conditions for 15 trials, as follows: HiIcPu, HiCoPu, HiIcPr, HiCoPr, LoIcPu, LoCoPu, LoIcPr, and LoCoPr; where “Hi” and “Lo” are higher (i.e., superior) and lower (i.e., inferior) hierarchies, “Ic” and “Co” indicate incongruent and congruent conditions, and “Pu” and “Pr” indicate public and private conditions, respectively. After completing all tasks, the participants filled out the questionnaires measuring individual differences, answered a few verbal interview questions, and then were debriefed about the deceptions regarding the absence of real human participants and the modified dictator game. The modified dictator game was included in the initial instructions to emphasize and enhance the external validity of the hierarchy manipulation, but it did not take place (Kim et al., 2021).

### Behavioral data analysis

Across all trials, preference ratings were coded in a binary fashion, with ratings of 3 (like) and 4 (strongly like) coded as 1 and ratings of 1 (strongly dislike) and 2 (dislike) as 0 because the changes across the category better represent a conforming behavior, which is changing one's belief or behavior to match that of others. Considering continuous changes would also be interesting, however, in that case, changes within a category would be treated the same as those across categories. For instance, changing one's preference rating from 1 (dislike very much) to 2 (dislike) and from 2 (dislike) to 3 (like) would both change 1 point on the Likert scale, but saying we dislike something less than we disliked it before may not be qualitatively the same as saying we like something that we disliked before. Then, the preference change in each trial was calculated using the absolute value of the difference between the participant's first and second preference ratings, which ranged from 0 to 1. For example, if a participant liked a given image when awarding the first preference rating (i.e., ratings of 3 or 4, coded 1) and disliked the same image when awarding the second preference rating (i.e., ratings of 1 or 2, coded 0), the preference change for this trial would be | 1 − 0 | = 1. The mean preference change score for each condition was then entered into a 2 (social hierarchy, superior and inferior) × 2 (observation, public and private) × 2 (partner's preference, incongruent and congruent) repeated-measures ANOVA (RM-ANOVA). Paired-sample *t* tests were performed to examine the simple main effects that contributed to statistically significant interaction effects. Moreover, we defined an individual tendency of strategic conformity (SC), that is, a greater likelihood of conforming to superior versus inferior partners' opinions under public versus private conditions, which was calculated as follows: [((HiIcPu – HiCoPu) – (LoIcPu – LoCoPu)) – ((HiIcPr – HiCoPr) – (LoIcPr – LoCoPr))], where the combination of the letters indicates the percentage of changing one's preference decision in the respective condition. All behavioral analyses were conducted using IBM SPSS Statistics (version 25) software. In addition, the graphs of the behavioral data were produced using GraphPad Prism software version 9.4.0 (Swift, 1997).

### fMRI data acquisition

All MR data were acquired using a 3.0T Siemens Magnetom Trio MRI scanner with a 12-channel head matrix coil located at the Korea University Brain Imaging Center. A high-resolution T1-weighted structural image was obtained [repeat time (TR) = 1900 ms; echo time (TE) = 2.52 ms; flip angle = 9; 256 × 256 matrix; 1 × 1 × 1 mm in-plane resolution]. After the anatomic scan, T2*-weighted functional images were acquired using a standard gradient-echo echoplanar imaging pulse sequence (TR = 2000 ms; TE = 30 ms; flip angle = 90°; field of view = 240 mm; 80 × 80 matrix; voxel size = 3 mm × 3 mm × 3 mm). Thirty-six interleaved axial slices with a 1 mm gap were acquired at an oblique angle to the anterior commissure–posterior commissure line to decrease the impact of susceptibility artifacts in the orbitofrontal cortex. The stimuli were presented through an MR-compatible liquid-crystal display (LCD) monitor mounted on a head coil (refresh rate, 85 Hz; display resolution, 800 × 600 pixels; viewing angle, 30° horizontal, 23° vertical). The participants used their right hand to respond via an MR-compatible four-button response box.

### Preprocessing of fMRI data

The fMRI data were preprocessed using SPM12 (Statistical Parametric Mapping; Wellcome Trust Center for Neuroimaging; http://www.fil.ion.ucl.ac.uk/spm/) in MATLAB 2019b. Before fMRI data preprocessing, the first five volumes (10 s) of each functional run were discarded to allow equilibration. All the scans were corrected for slice timing and head motion. After realignment, functional data were normalized to the Montreal Neurologic Institute (MNI) template (resampling voxel size, 2 × 2 × 2 mm^3^) and spatially smoothed with a Gaussian kernel of 8 mm FWHM.

### fMRI data statistical analysis

#### First-level general linear model analysis

A first-level general linear model (GLM) was constructed to estimate neural responses to the experimental conditions. The GLM included 16 regressors of interest consisting of (1) the first rating of all images with the participant's response time as a boxcar function; (2) four regressors representing the other-feedback onset of the partner's hierarchy and preference for each condition (i.e., HiCo, HiIc, LoCo, and LoIc); (3) eight regressors representing the observation onset for each condition (i.e., HiIcPu, HiCoPu, HiIcPr, HiCoPr, LoIcPu, LoCoPu, LoIcPr, LoCoPr); (4) two regressors representing the onset of the second rating presentation, with a parametric modulator reflecting choice behavior (switch or stay, which equals 1 if the participant conforms to partner's opinion and −1 otherwise); and (5) the onset of decision. Additionally, six motion parameters were included as regressors of no interest. Specifically, the decision phase with a button press was added to the GLM as a regressor to reduce any noise associated with the motion of pressing the button. All regressors of interest were convolved with a canonical hemodynamic response function.

#### Second-level group analysis

Subject-specific contrast images were entered into a second-level analysis using a one-sample *t* test. We hypothesized that the participants would establish a strategy for preference change when their partner's hierarchy and preference were displayed. To test this, we defined the contrast of the social hierarchy × partner preference interaction and tested the group effect to find the region that responded to the partner's hierarchy and preference information when it was presented. Consistent with the behavioral data analysis, we also defined the contrast of the three-way interaction [i.e., ((HiIcPu – HiCoPu) – (LoIcPu – LoCoPu)) – ((HiIcPr – HiCoPr) – (LoIcPr – LoCoPr))]. In addition, based on the behavioral data, we aimed to identify the region that responded to the interaction of partner preference × observation in the superior condition and the interaction of social hierarchy × observation in the incongruent condition at the time of observation cue display. To compare the neural activity levels, beta estimates were extracted and entered into a 2 × 2 RM-ANOVA. In addition, contrast images related to the switching current preference (i.e., Switch > Stay) were entered into a second-level analysis. Moreover, voxelwise multiple regression analysis was used to identify the brain regions whose activities correlated with individual SC scores. Individual contrast maps constructed for the second-level group analysis were regressed on individual SC scores. The second-level maps, behavioral data, and extended table, which include all significant peak voxels, have been made publicly available through the Open Science Framework and can be accessed at https://osf.io/5ds3p/.

#### Statistical thresholding

The resulting statistical maps were thresholded at *p* < 0.05, using whole-brain correction with a false discovery rate (FDR) for multiple comparisons. We used the MarsBaR toolbox (Brett et al., 2002; https://marsbar-toolbox.github.io/) to extract beta estimates from the regions and visualize a significant interaction effect pattern. Neural data were plotted and graphed using GraphPad Prism software version 9.4.0 (Swift, 1997).

## Results

### Behavioral results

We aimed to investigate whether and how individual preferences can be changed to align with those of people in a higher social hierarchy, particularly in public situations. In the behavioral task, participants were presented with a series of fractal images and asked to rate how much they liked or disliked them. They then viewed the ratings from their partners for the same images and were asked to finalize their ratings. The social hierarchy of the two partners was manipulated before the behavioral task, so that one of them was inferior and the other was superior to the participant. The participants were also informed that their final ratings would be reported to their partners in half the trials (public condition) but not in the other half (private condition; Fig. 1). To quantify the change in preference in each trial, we calculated the absolute value of the difference between the participant's first and second preference ratings. For example, preference changes were coded in a binary fashion, conforming to others was coded as 1, and nonconformity was coded as 0.

The mean preference change score for each condition was then entered into a 2 (social hierarchy, superior and inferior) × 2 (observation, public and private) × 2 (partner's preference, incongruent and congruent) RM-ANOVA. The RM-ANOVA results indicated that the changes in participants' preferences regarding the fractal images were influenced by the social hierarchy of the partner (*F*_(1,44)_ = 42.93, *p* < 0.001, $\eta_{p}^{2}$ = 0.49), observation (*F*_(1,44)_ = 14.96, *p* < 0.001, $\eta_{p}^{2}$ = 0.25), and the partner's preference (*F*_(1,44)_ = 58.68, *p* < 0.001, $\eta_{p}^{2}$ = 0.57). Moreover, this analysis yielded significant two-way interaction effects of social hierarchy × observation (*F*_(1,44)_ = 13.07, *p* = 0.001, $\eta_{p}^{2}$ = 0.2), social hierarchy × partner's preference (*F*_(1,4)_ = 35.91, *p* < 0.001, $\eta_{p}^{2}$ = 0.45), and observation × partner's preference (*F*_(1,4)_ = 20.68, *p* < 0.001, $\eta_{p}^{2}$ = 0.32). A significant three-way interaction effect on strategic conformity was also significant (*F*_(1,44)_ = 24.57, *p* < 0.001, $\eta_{p}^{2}$ = 0.36).

In addition to analyzing the participants' choices in a binary fashion, we conducted an additional analysis treating them as a continuous variable. Specifically, the change in preference ratings was calculated by the absolute value of the difference between the participant's second and first ratings. For example, if a participant's first rating is 2, the change would be calculated as follows: | 1 − 2 | = 1 for a change from 2 − 1, | 2 − 2 | = 0 for no change, | 3 − 2 | = 1 for a change from 2 − 3, and | 4 − 2 | = 2 for a change from 2 − 4. The mean preference change score for each condition was then included in a 2 (social hierarchy, superior and inferior) × 2 (observation, public and private) × 2 (partner's preference, incongruent and congruent) repeated-measures ANOVA. The results indicated a significant three-way interaction effect (*F*_(1,44)_ = 23.82, *p* < 0.001, $\eta_{p}^{2}$ = 0.35). Moreover, this analysis yielded significant two-way interaction effects of social hierarchy × observation (*F*_(1,44)_ = 10.35, *p* = 0.002, $\eta_{p}^{2}$ = 0.19), social hierarchy × partner's preference (*F*_(1,44)_ = 31.22, *p* < 0.001, $\eta_{p}^{2}$ = 0.42), and observation × partner's preference (*F*_(1,44)_ = 10.91, *p* = 0.002, $\eta_{p}^{2}$ = 0.20). Additionally, the changes in participants' preferences were influenced by the social hierarchy of the partner (*F*_(1,44)_ = 30.43, *p* < 0.001, $\eta_{p}^{2}$ = 0.41), the observation condition (*F*_(1,44)_ = 13.47, *p* < 0.001, $\eta_{p}^{2}$ = 0.23), and the partner's preference (*F*_(1,44)_ = 52.56, *p* < 0.001, $\eta_{p}^{2}$ = 0.54). Importantly, the results obtained using the continuous variable were similar to those obtained using the binary variable, but we chose the binary fashion to extend our previous study (Kim et al., 2021).

**Figure 2.**
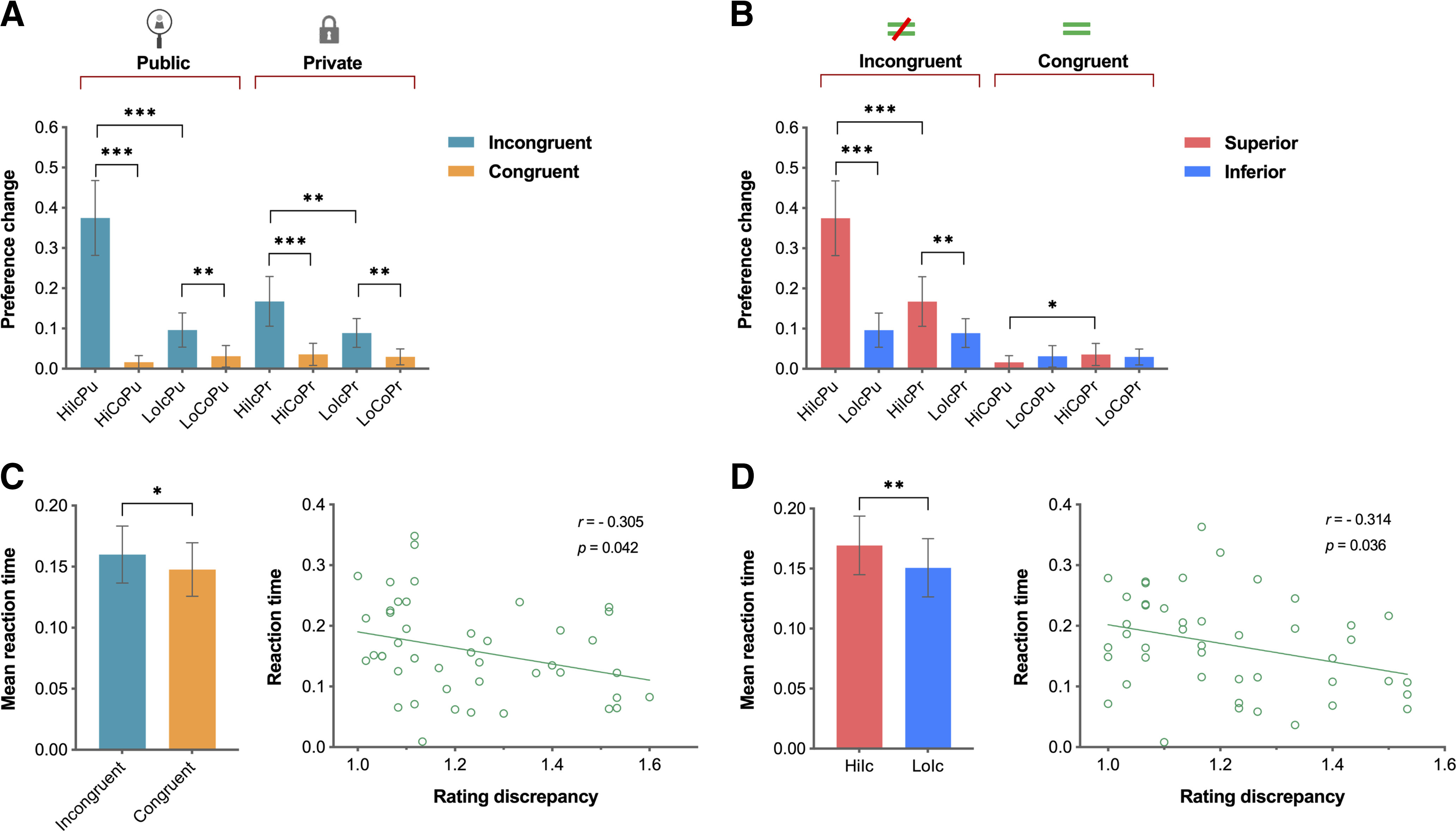
Behavioral data analysis revealed that for public versus private trials, participants were more likely to change their preferences to match those of the superior versus inferior partner in the incongruent versus congruent condition. ***A***, ***B***, The behavioral results are shown separately for the public and private trials (***A***) as well as for the incongruent and congruent preference trials (***B***). ***C***, The reaction times were significantly longer for incongruent trials compared with congruent trials, and a significant negative correlation was observed between the rating discrepancy and the reaction times in the incongruent conditions. ***D***, Within the incongruent trials, the reaction times were significantly longer when participants were presented with superiors' preferences compared with inferiors' preferences, and there was a significant negative correlation between the rating discrepancy and the reaction times in the superior conditions. Error bars indicate 95% confidence intervals; *p < 0.05, **p < 0.01, ***p < 0.001. Hi = superior; Lo = inferior; Ic = incongruent; Co = congruent; Pu = public; Pr = private.

Next, *post hoc* paired-sample *t tests* were conducted to assess the behavioral responses between each pair of conditions. In the public conditions, participants were more likely to change their preferences in line with those of the superior (mean = 0.38, SD = 0.31) than the inferior partner (mean = 0.10, SD = 0.14) in the incongruent condition (*t*_(44)_ = 6.27, *p* < 0.001, Cohen's *d* = 1.16). On the other hand, there was no significant difference between inferiors' (mean = 0.03, SD = 0.09) and superiors' (mean = 0.02, SD = 0.06) decisions in the congruent condition (*t*_(44)_ = 1.75, *p* = 0.086, Cohen's *d* = 0.13). Furthermore, participants were more likely to change their preference following the superior's opinion in the incongruent condition (mean = 0.38, SD = 0.31) than in the congruent condition (mean = 0.17, SD = 0.21; *t*_(44)_ = 7.60, *p* < 0.001, Cohen's *d* = 0.79), and they were also more likely to change their preference following the inferior's opinion in the incongruent condition (mean = 0.10, SD = 0.14) than in the congruent condition (mean = 0.03, SD = 0.09; *t*_(44)_ = 3.13, *p* = 0.003, Cohen's *d* = 0.60; Fig. 2*A*).

In the private conditions, participants were more inclined to conform to the superior (mean = 0.17, SD = 0.21) than to the inferior (mean = 0.09, SD = 0.12) in the incongruent condition (*t*_(44)_ = 3.23, *p* = 0.002, Cohen's *d* = 0.47), but there was no significant difference in preference rating changes between superior (mean = 0.04, SD = 0.09) and inferior (mean = 0.03, SD = 0.07) trials in the congruent condition (*t*_(44)_ = 0.55, *p* = 0.585, Cohen's *d* = 0.12). Moreover, participants were more likely to change their preference following the superior in the incongruent (mean = 0.17, SD = 0.21) than the congruent condition (mean = 0.04, SD = 0.09; *t*_(44)_ = 4.74, *p* < 0.001, Cohen's *d* = 0.81), and they were also more likely to change their preference following the inferior in the incongruent (mean = 0.09, SD = 0.11) than in the congruent condition (mean = 0.03, SD = 0.07; *t*_(44)_ = 3.70, *p* = 0.001, Cohen's *d* = 0.65; Fig. 2*A*).

Moreover, *post hoc* paired-sample *t tests* indicated that participants were more likely to consider information on social hierarchy and observations in the incongruent condition. In the incongruent conditions, participants were more likely to change their preference under the public (mean = 0.38, SD = 0.31) than under the private condition (mean = 0.17, SD = 0.21) in the superior (*t*_(44)_ = 4.57, *p* < 0.001, Cohen's *d* = 0.79). They were more likely to change their decision in line with those of the superior (mean = 0.38, SD = 0.31) rather than the inferior partner (mean = 0.10, SD = 0.14) under the public condition (*t*_(44)_ = 6.27, *p* < 0.001, Cohen's *d* = 1.16). Furthermore, participants were more likely to conform to the superior's opinion (mean = 0.17, SD = 0.21) than the inferior's opinion (mean = 0.09, SD = 0.12) under the private condition (*t*_(44)_ = 3.23, *p* = 0.002, Cohen's *d* = 0.47; Fig. 2*B*). For the congruent conditions, participants were more likely to change their preference under the private (mean = 0.04, SD = 0.09) than under the public condition (mean = 0.02, SD = 0.06), when the superior's rating was presented (*t*_(44)_ = 2.67, *p* = 0.011, Cohen's *d* = 0.26; Fig. 2*B*). We further examined the difference between the baseline (i.e., zero) and the preference changes in the congruent conditions. Participants were more likely to change their preferences under the private conditions compared with the baseline when the superior's rating was presented (*t*_(44)_ = 2.60, *p* = 0.013) and when the inferior's rating was presented (*t*_(44)_ = 3.01, *p* = 0.004). Additionally, participants were more likely to change their preferences under the public conditions compared with the baseline when the inferior's rating was presented (*t*_(44)_ = 2.33, *p* = 0.024), whereas there was no significant difference when the superior's rating was presented (*t*_(44)_ = 1.97, *p* = 0.055).

Additionally, we examined reaction time and the rating discrepancy to investigate whether the participants' conforming behaviors are a value-based (i.e., cost-benefit computation) or the rule-based decision. Participants displayed longer reaction times in the incongruent conditions than in the congruent conditions (*t*_(44)_ = 2.08, *p* = 0.044, Cohen's *d* = 0.16; Fig. 2*C*). Moreover, within the incongruent trials, reaction times were significantly longer when participants were presented with superiors' preferences compared with inferiors' preferences (*t*_(44)_ = 2.74, *p* = 0.009, Cohen's *d* = 0.22; Fig. 2*D*).

To evaluate rating discrepancy across incongruent trials, we assigned a value of 1 when participants initially chose a rating of 2 (dislike) or 3 (like), and a value of 2 when participants initially chose a rating of 1 (strongly dislike) or 4 (strongly like). Among the incongruent trials, we discovered a significant negative correlation between rating discrepancy and reaction time (*r*_(45)_ = −0.305, *p* = 0.042, two-tailed Pearson correlation; Fig. 2*C*). However, no significant correlation was observed between rating discrepancy and reaction time in the congruent conditions (*r*_(45)_ = −0.246, *p* = 0.103, two-tailed Pearson correlation). Notably, a significant negative correlation was observed between rating discrepancy and reaction time in the superior conditions (*r*_(45)_ = −0.321, *p* = 0.032, two-tailed Pearson correlation; Fig. 2*D*). However, no significant correlation was observed in the inferior conditions (*r*_(45)_ = −0.225, *p* = 0.137, two-tailed Pearson correlation).

Furthermore, our study revealed that participants made choices with a large rating discrepancy (initial rating of 1 or 4) 25% of the time in the superior conditions (336 of 1350 choices), and 12% of those choices exhibited conforming behavior (39 of 336 choices). Conversely, they made choices with a small rating discrepancy (initial rating 2 or 3) 75% of the time in the superior conditions (1014 of 1350 choices), and 32% of those choices exhibited conforming behavior (327 of 1014 choices). In other words, participants were more likely to change their preferences when the rating discrepancy was small, and the superior's rating was presented (*t*_(44)_ = 5.72, *p* < 0.001, Cohen's *d* = 0.86).

Based on the results of the correlation analysis, participants seemed capable of swiftly deciding not to agree with the superior's opinions when the rating discrepancy was large. However, an additional cognitive process may be required to compare their preferences with those of the superior when the rating discrepancy was small. To summarize, these findings suggest that participants experienced heightened conflict in the superior conditions when the rating discrepancy was smaller, indicating that their conforming behaviors were not solely based on simple rule-based decision but rather on complex value-based decision-making, considering various factors such as the disparity between their initial choices and those of others.

Finally, considering the previous study's findings (Kim et al., 2021), which indicated that individual differences in social dominance orientation (SDO) and fear of negative evaluation (FNE) can predict the extent of social conformity, we conducted correlation analyses to assess the relationships between each measurement (i.e., the SDO and FNE) and individual SC scores. The results of the correlation analyses demonstrated a statistically significant positive correlation between FNE and SC scores (*r*_(45)_ = 0.252, *p* = 0.048, one-tailed Pearson correlation). However, no statistically significant positive correlation was observed between SDO scores and SC scores (*r*_(45)_ = 0.079, *p* = 0.302, one-tailed Pearson correlation).

### Neuroimaging results

#### Neural regions responding to congruency with a superior partner's opinion

**Figure 3.**
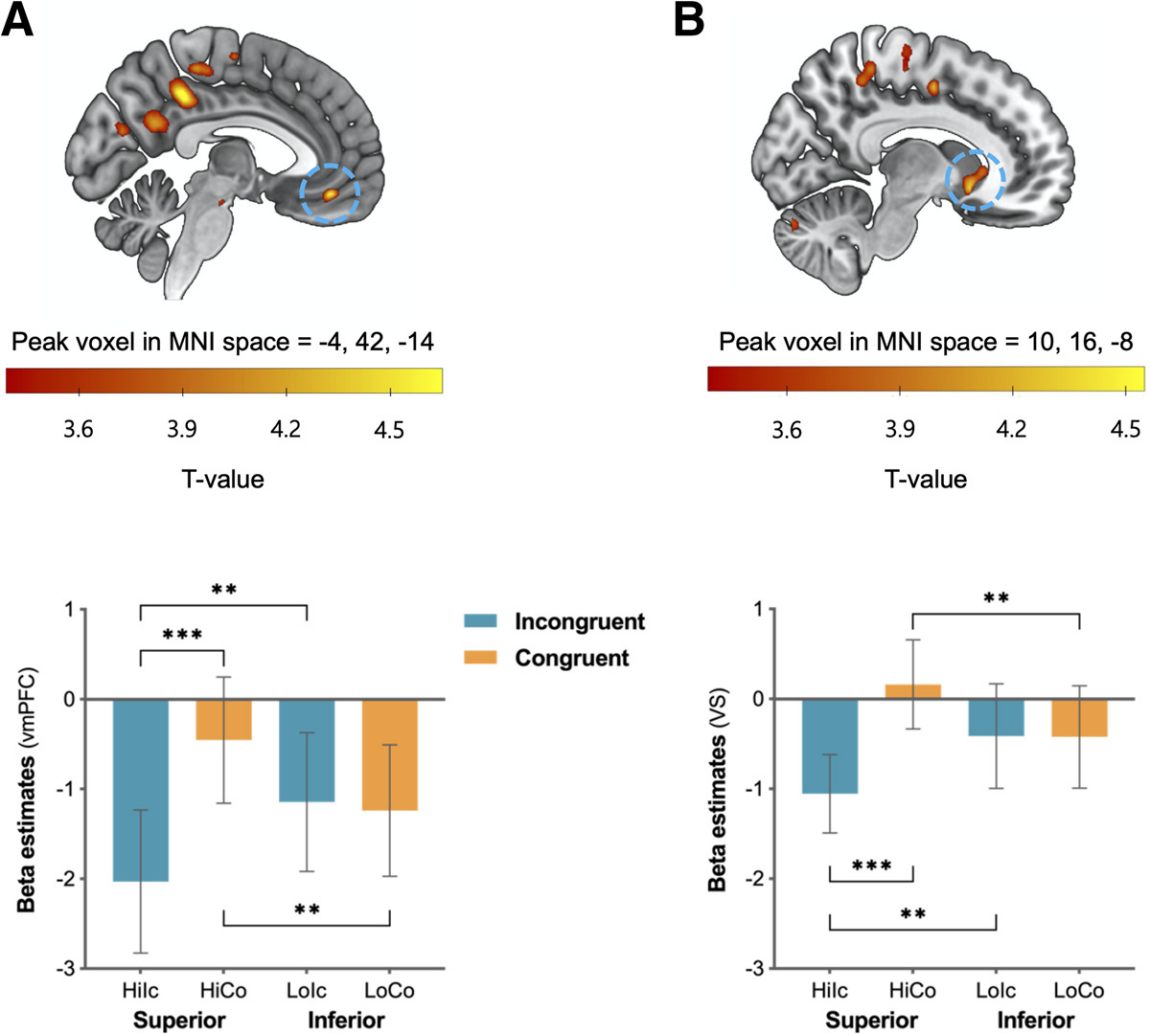
***A***, ***B***, The vmPFC (***A***) and the VS (***B***) activation showing the interaction effect of social hierarchy × partner's preference. Error bars indicate 95% confidence intervals; **p < 0.01, ***p < 0.001. Hi = superior; Lo = inferior; Ic = incongruent; Co = congruent.

First, we investigated the main effect of social hierarchy at the other-feedback onset but found any statistically significant clusters either in the contrast of superior versus inferior conditions or in the reverse contrast. We then aimed to identify neural regions that respond differentially to conflicting opinions depending on the social hierarchy by examining the contrast maps of social hierarchy (i.e., superior vs inferior) × partner's preference (i.e., congruent vs incongruent) interaction [i.e., (HiCo – HiIc) – (LoCo – LoIc)] at the time of other-feedback display. Significant activity was found in the vmPFC (*F*_(1,44)_ = 16.65, *p* < 0.001, $\eta_{p}^{2}$ = 0.28; peak coordinates = –4, 42, –14; FDR, *p* = 0.043; Fig. 3*A*, Table 1) and the VS (*F*_(1,44)_ = 19.41, *p* < 0.001, $\eta_{p}^{2}$ = 0.31; peak coordinates = 10, 16, –8; FDR, *p* = 0.043; Fig. 3*B*, Table 1). *Post hoc* paired-sample *t* tests with the mean beta estimates from the two regions indicated that activation was significantly greater when participants' preferences were congruent with those of the superior compared with the inferior partners both in the vmPFC (*t*_(44)_ = 3.07, *p* = 0.004) and in the VS (*t*_(44)_ = 2.95, *p* = 0.005). These two regions exhibited greater responses when participants' opinions were incongruent with those of the inferior compared with the superior partners (vmPFC, *t*_(44)_ = 2.87, *p* = 0.006; VS, *t*_(44)_ = 2.71, *p* = 0.009) and also when their preferences were congruent versus incongruent with those of the superior partner (vmPFC, *t*_(44)_ = 5.37, *p* < 0.001; VS, *t*_(44)_ = 5.66, *p* < 0.001). Given that the vmPFC and the VS are key components of the reward circuitry in the brain, these results suggest that participants experience heightened reward when their preferences are congruent with those of their superiors but experience reduced reward when their preferences are incongruent.

**Table 1.** Significantly activated (*p* < 0.05, FDR corrected) brain regions

Brain regions	Left/right (L/R)	Cluster size (voxels)	Peak MNI coordinates			*t* statistic
			*x*	*y*	*z*	
Social hierarchy × partner's preference						
vmPFC	L	97	−4	42	−14	4.65
VS	R	75	10	16	−8	4.54
Observation × partner's preference in the superior condition						
Superior frontal gyrus	R	2094	18	36	44	5.90
dmPFC	L		−4	54	28	5.57
vmPFC	L	360	−2	28	−16	5.79
Voxelwise multiple regression: social hierarchy × partner's preference						
rmPFC	L	447	−14	54	−6	5.73
Voxelwise multiple regression: social hierarchy × observation in the incongruent condition						
Middle frontal gyrus	L	3069	−36	50	−8	4.22
dmPFC	R		6	24	46	3.82
Paracentral lobule	L	6626	−6	−30	56	5.01
TPJ	R		60	−44	28	4.34

#### Neural regions responding differently to conflicting opinions from superior partner depending on the context of observation

**Figure 4.**
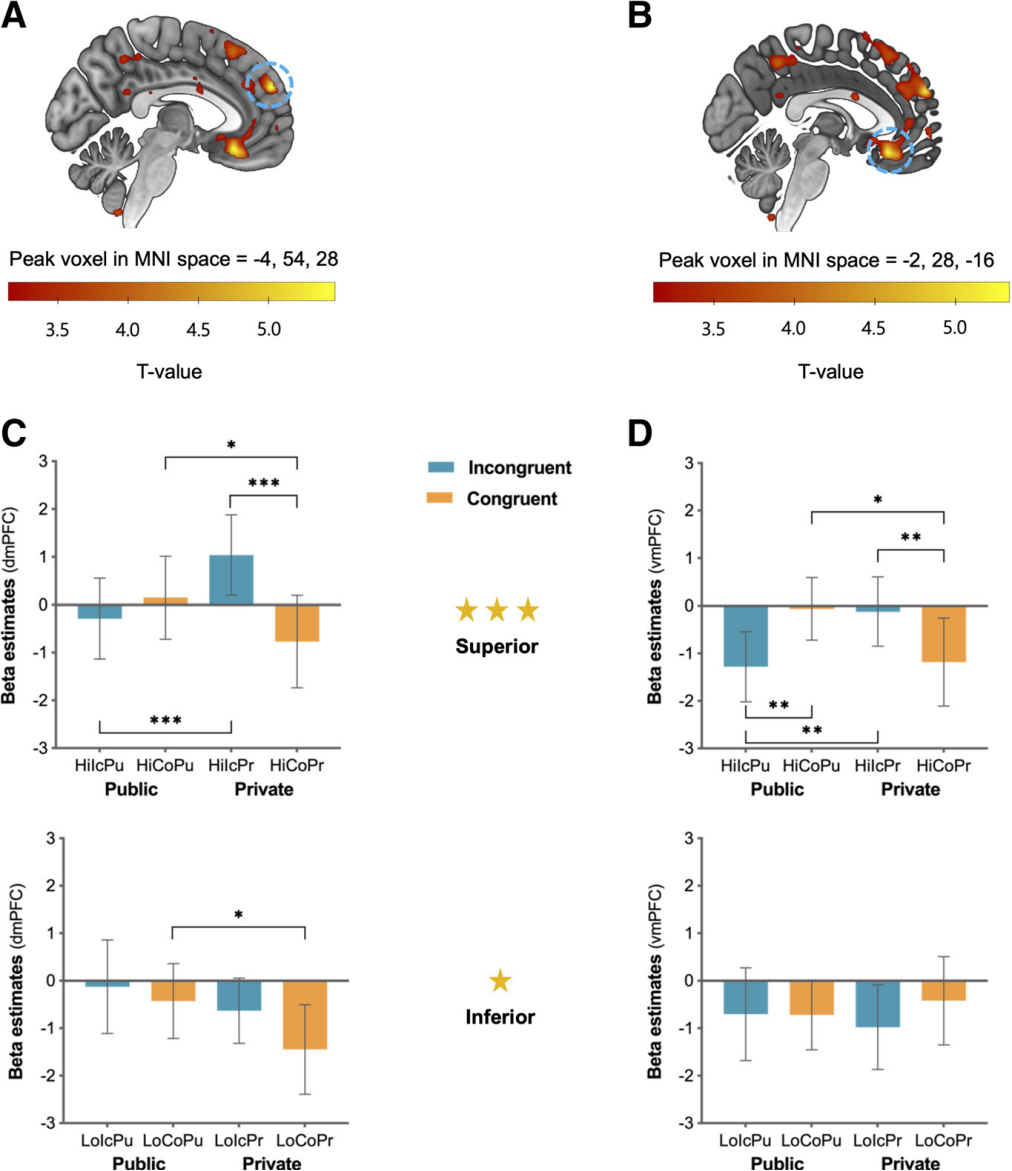
***A–D***, The dmPFC (***A***, ***C***) and the vmPFC (***B***, ***D***) activation showing the interaction effect of observation × partner's preference in the superior condition. Error bars indicate 95% confidence intervals; *p < 0.05, **p < 0.01, ***p < 0.001. Hi = superior; Lo = inferior; Ic = incongruent; Co = congruent; Pu = public; Pr = private.

Next, we aimed to identify the neural regions associated with increased conformity to the social hierarchy in the public versus the private condition by examining the contrast maps at the time of observation cue display. Based on the main behavioral results, we first examined the contrast of the three-way interaction but found no significant clusters surviving the threshold. Given that the three-way interaction effect was driven mainly by superior conditions, we focused on the contrast of observation × partner's preference interaction in the superior condition [i.e., (HiIcPu – HiCoPu) – (HiIcPr – HiCoPr)] and found significant clusters in the dmPFC (*F*_(1,44)_ = 32.54, *p* < 0.001, $\eta_{p}^{2}$ = 0.43; peak coordinates = –4, 54, 28; FDR, *p* = 0.013; Fig. 4*A*, Table 1) and the vmPFC (*F*_(1,44)_ = 27.51, *p* < 0.001, $\eta_{p}^{2}$ = 0.39; peak coordinates = –2, 28, –16; FDR, *p* = 0.013; Fig. 4*B*, Table 1). *Post hoc* analyses revealed that dmPFC activation was significantly greater when participants' preferences were incongruent versus congruent (*t*_(44)_ = 3.54, *p* < 0.001) in the private condition and when participants' preferences were incongruent in the private versus public condition (*t*_(44)_ = 3.38, *p* = 0.002; Fig. 4*C*). Interestingly, the vmPFC showed increased activity when participants' preferences were incongruent versus congruent in the private condition (*t*_(44)_ = 2.50, *p* = 0.016; Fig. 4*D*). Unlike the dmPFC, vmPFC activity showed a greater increase when participants' preferences were congruent versus incongruent in the public condition (*t*_(44)_ = 2.87, *p* = 0.006). Additionally, activation in the vmPFC was significantly greater when participants' preferences were congruent in the public condition than in the private condition (*t*_(44)_ = 2.36, *p* = 0.023) and when their preferences were incongruent in the private condition than in the public condition (*t*_(44)_ = 3.05, *p* = 0.004). In sum, these results suggest that both the vmPFC and the dmPFC are involved in the additional cognitive processes associated with strategic conformity, particularly when participants' preferences are incongruent with those of their superior partners in the private condition.

For completeness, we also tested whether the neural signals in the dmFC and vmPFC in the superior condition were involved in differentiating social information in the inferior condition. Similar to the analyses for the superior condition, we examined the interaction contrast of observation (i.e., public versus private condition) × partner's preference in the inferior condition [i.e., (LoIcPr – LoCoPr) – (LoIcPu – LoCoPu)] but found no significant interaction effect in either region. The dmPFC only exhibited a greater response when the opinions of the inferior partner were congruent in the public versus the private condition (*t*_(44)_ = 2.14, *p* = 0.038; Fig. 4*C*).

#### Neural signatures of individual differences in strategic conformity to the social hierarchy (at the time of other-feedback display)

**Figure 5.**
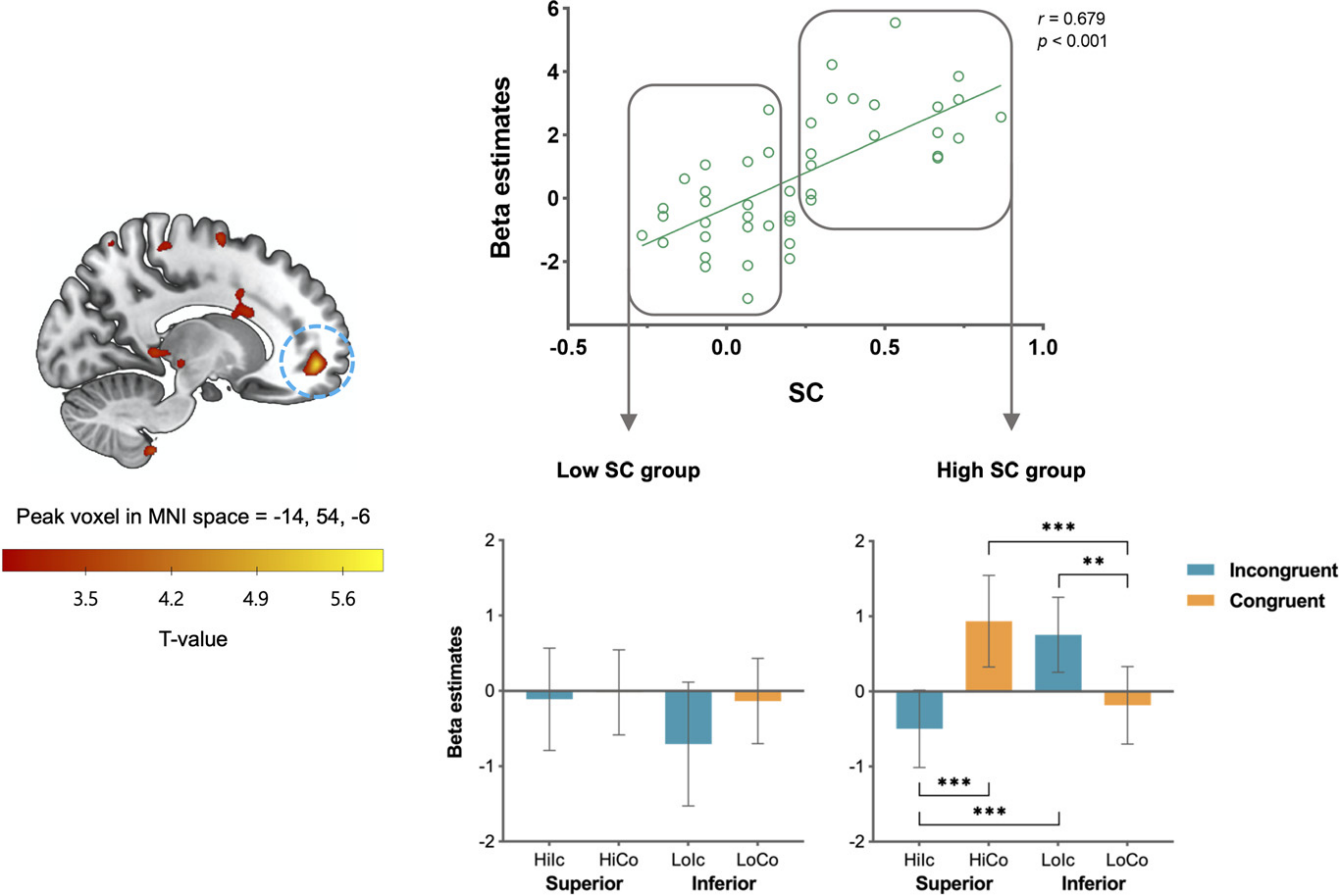
The voxelwise multiple regression analysis revealed activation in the rmPFC is positively correlated with individual participants' SC scores. The rmPFC activity in the high SC group was significantly greater when participants' preferences were congruent versus incongruent with those of the superior compared with the inferior partners. Error bars indicate 95% confidence intervals; **p < 0.01, ***p < 0.001. Hi = superior; Lo = inferior; Ic = incongruent; Co = congruent.

We found a wide range of individual differences in the degree of conformity to the social hierarchy in the public versus private condition (i.e., strategic conformity or SC score). To identify the neural signatures of such individual differences, we ran a voxelwise multiple regression analysis and found a large cluster in the rmPFC (peak coordinates = –14, 54, –6; FDR, *p* = 0.019; Fig. 5, Table 1), showing a significant positive correlation between the SC scores and the contrast maps of social hierarchy × partner's preference interaction at the time of other-feedback display. To see more precisely how SC modulates the social hierarchy × partner preference interaction effect in the rmPFC, we divided participants into high (mean = 0.51, SD = 0.20, *n* = 19) and low (mean = −0.03, SD = 0.12, *n* = 21) SC groups using a median split; the median group (*n* = 5) was excluded from subsequent analyses. In the high SC group, the rmPFC activation was significantly greater when participants' preferences were congruent versus incongruent with those of the superior partner, but this pattern was reversed for the inferior partner, showing a significant two-way interaction effect (*F*_(1,18)_ = 55.63, *p* < 0.001, $\eta_{p}^{2}$ = 0.76). *Post hoc t* tests indicated that the rmPFC activation in the high SC was significantly greater when participants' preferences were congruent with those of the superior compared with the inferior partners (*t*_(18)_ = 4.74, *p* < 0.001) and also when partners' preferences were congruent versus incongruent with those of the superior partner (*t*_(18)_ = 4.90, *p* < 0.001). It should be noted that the rmPFC activity was significantly greater when participants' opinions were incongruent with those of the inferior compared with the superior partners (*t*_(18)_ = 7.50, *p* < 0.001) and also when participants' preferences were incongruent versus congruent with those of the inferior partner (*t*_(18)_ = 4.22, *p* = 0.001). Unlike the high SC group, the low SC group showed no significant two-way interaction effect (*F*_(1,20)_ = 2.50, *p* = 0.129, $\eta_{p}^{2}$ = 0.11) and no significant difference in any pairs of conditions. The current findings suggest that activity in the rmPFC reflects the additional cognitive processes of considering others' hierarchical positions and the congruence of opinions to compute the value of decision-making aimed at conforming to superiors.

#### Neural signatures of individual differences in strategic conformity to the social hierarchy (at the time of observation cue display)

**Figure 6.**
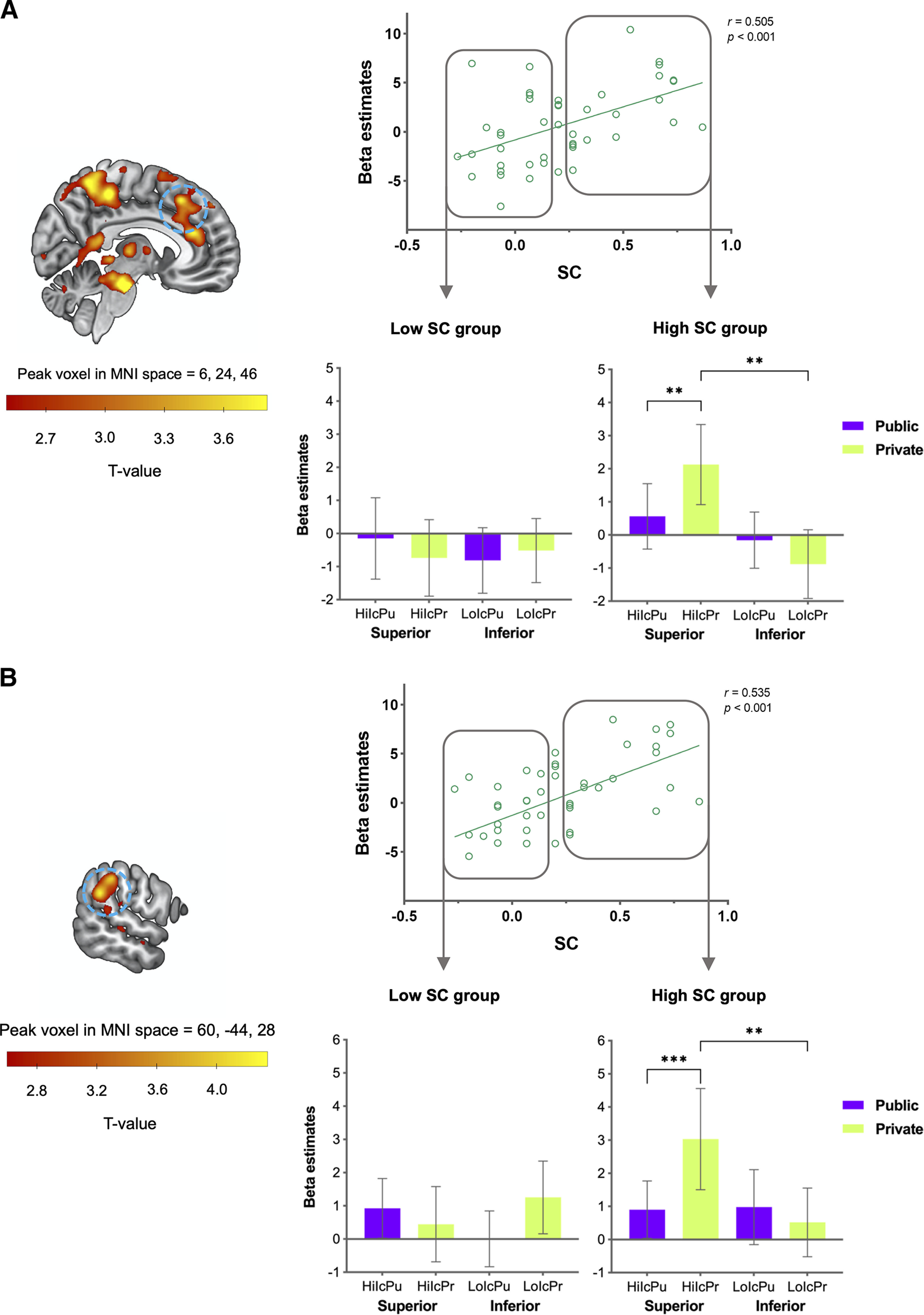
***A***, ***B***, The voxelwise multiple regression analysis showed the dmPFC (***A***) and TPJ (***B***) activities were positively correlated with individual participants' SC scores. These two regions in the high SC group were significantly greater when participants' decisions were under the private versus public condition with those of the superior compared with the inferior partners. Error bars indicate 95% confidence intervals; **p < 0.01, ***p < 0.001. Hi = superior; Lo = inferior; Ic = incongruent; Co = congruent; Pu = public; Pr = private.

Next, we aimed to identify the neural signatures of individual differences in the degree of conformity to the social hierarchy in the public versus private conditions when the observation cue was displayed. This analysis revealed that the dmPFC (peak coordinates = 6, 24, 46; FDR, *p* = 0.031; Fig. 6*A*, Table 1) and TPJ (peak coordinates = 60, −44, 28; FDR, *p* = 0.03, Fig. 6*B*, Table 1) activities were positively correlated with SC scores in the contrast of the social hierarchy × observation interaction. *Post hoc* analyses demonstrated that the high SC group showed significantly greater activities in both regions in the private versus public condition when paired with the superior versus inferior partners (dmPFC, *F*_(1,18)_ = 7.14, *p* = 0.016, $\eta_{p}^{2}$ = 0.28; TPJ, *F*_(1,18)_ = 9.27, *p* = 0.007, $\eta_{p}^{2}$ = 0.34). The two regions showed significantly greater activity in the private condition when paired with superior and inferior partners (dmPFC, *t*_(18)_ = 3.36, *p* = 0.003; TPJ, *t*_(18)_ = 2.95, *p* = 0.009) and a greater response when paired with a superior partner in the public versus private condition (dmPFC, *t*_(18)_ = 3.13, *p* = 0.006; TPJ, *t*_(18)_ = 4.33, *p* < 0.001). On the other hand, the low SC group showed no significant interaction between social hierarchy × observation (dmPFC, *F*_(1,20)_ = 1.10, *p* = 0.314, $\eta_{p}^{2}$ = 0.05; TPJ, *F*_(1,20)_ = 3.00, *p* = 0.099, $\eta_{p}^{2}$ = 0.13), showing no significant differences between any pair of conditions. The present findings suggest that increased activity in the dmPFC and the TPJ are implicated in the additional cognitive processes of considering others' hierarchical positions and the observational context, facilitating the detection and resolution of conflicts in the private condition.

## Discussion

In the present study, we aimed to identify the neural mechanisms underlying the increased conformity to superior versus inferior others in an observational context. Replicating our previous study (Kim et al., 2021), we showed that participants were more likely to conform to others in higher versus lower social positions, especially when participants believed that their decisions were visible to those in higher social positions. In the congruent conditions, participants demonstrated a propensity to retain their opinions especially when their decisions were visible to the superior partner, potentially mirroring the motivation underlying the conforming behavior observed in the incongruent conditions. In addition, consistent with our hypothesis, we disentangled the functionally distinct contributions of the mPFC subregions to increased conformity to social hierarchy under social observation. First, the vmPFC, along with the VS, showed increased activities when participants' preferences were congruent versus incongruent with those of the superior compared with the inferior partners, regardless of behavioral manifestation. Second, the rmPFC showed increased activity when one's initial decision was congruent with that of a superior partner and incongruent with that of an inferior partner, revealing its goal-dependent valuation. Third, the dmPFC, along with the TPJ, showed increased activity in the private versus public condition when paired with superior versus inferior partners. However, unlike the vmPFC, such activity patterns in the rmPFC and dmPFC were observed only among those with a higher tendency to conform to the social hierarchy under social observation. The present findings are consistent with a recent mPFC model for social valuation, in which the ventral-to-dorsal gradient of the mPFC subregions is hierarchically structured so that it exerts increasing allostatic control over internalized social responses by adding more information from the external environment (Kim, 2020).

### Functionally dissociable roles of the mPFC subregions in strategic social conformity

In social decision-making, people are usually guided by the opinions, preferences, and attitudes of the majority (Klucharev et al., 2009; Izuma and Adolphs, 2013) or others with a higher social position (Deutsch and Gerard, 1955; Cialdini and Goldstein, 2004; Galinsky et al., 2008; Hays and Goldstein, 2015; Qi et al., 2018; Kim et al., 2021; Li et al., 2021). Several previous neuroimaging studies have suggested that such a social phenomenon may be closely related to the function of the mPFC (Klucharev et al., 2009; Campbell-Meiklejohn et al., 2010; Zaki et al., 2011; Izuma and Adolphs, 2013; Kumaran et al., 2016; Ligneul et al., 2016; Wu et al., 2016; Li et al., 2021).

The vmPFC, working in conjunction with the VS as the two key neural hubs of the reward processing network, has often been associated with social conformity, supporting the idea that conformity to others can be viewed as a type of reward-seeking behavior (Baumeister and Leary, 1995; Cialdini and Goldstein, 2004; Klucharev et al., 2009; Campbell-Meiklejohn et al., 2010; Zaki et al., 2011; Ruff and Fehr, 2014). Consistent with this view, the vmPFC and VS activities increased when one's initial choice was congruent versus incongruent with those of superior others and decreased when one's initial choice was incongruent with those of superior versus inferior partners across all participants, regardless of whether they conformed to the superior partners' opinions. These results suggest that people generally have a highly internalized reward value associated with social hierarchy (Zink et al., 2008), which can lead to a sense of gratification when they find their opinions congruent with those of superior, but not inferior, others. Such an internalized reward value of social hierarchy may serve as a major driving force for subsequent conformity to a superior partner. Considering previous findings that demonstrate the modulatory role of vmPFC activity in visual attention (Lim et al., 2011), future research using eye-tracking techniques would be valuable in investigating the extent to which the vmPFC response to a superior's opinion is related to the allocation of visual attention to the rank or rating information of the other person.

Unlike the vmPFC, the rmPFC, located immediately dorsal to the vmPFC, appears to be more closely tied to actual conformity behaviors. The rmPFC showed increased activity when an initial decision was congruent with a superior partner and incongruent with an inferior partner only among individuals with a strong tendency toward SC (i.e., those with higher SC). Previous studies have shown that the rmPFC tracks and updates information about the social hierarchy (Kumaran et al., 2016; Ligneul et al., 2016; Qu et al., 2017) and promotes self-enhancement behavior in a context-dependent manner (Izuma et al., 2010; Hoorn et al., 2016; Jung et al., 2018; Yoon et al., 2018, 2021; Kim and Kim, 2021). The present findings suggest that rmPFC activity reflects additional consideration of social hierarchy and congruency of opinions to compute the value of the decision to conform to superiors as a means to promote one's relative social position.

The dmPFC, along with the TPJ, showed increased activity in the private versus public condition when paired with superior versus inferior partners. As seen above, the rmPFC can compute the value of the decision to conform to a superior partner even before a social observation cue appears. Thus, conforming decisions become default decisions when the public condition cue is displayed, whereas extra control is required to override the default decision when the private condition cue is displayed. In other words, in the private condition, people with higher SC scores would face a stronger conflict between the desire to earn a favorable impression from others with a high-ranking position (Kim et al., 2021) and the desire to maintain their initial opinion for consistency (Cialdini et al., 1995; Gawronski, 2012). Therefore, increased activity in the dmPFC and TPJ in the private condition may be involved in detecting and resolving the conflict through more careful consideration of external information, such as more details of the visual features of the images being evaluated, as well as the mentalizing process (Frith and Frith, 2006; Hampton et al., 2008; Shenhav et al., 2016; Kim, 2020; Konovalov et al., 2021).

### Strategic conformity as a means to minimize the metabolic cost of the body

According to several previous theoretical works, human social behaviors can be best understood as products of the neural process of seeking optimal strategies to minimize prediction errors arising from the mismatch between the internal model of the world and actual bodily consequences and to regulate one's interaction with the social environment in a metabolically efficient way (Constant et al., 2019; Theriault et al., 2021). One representative example of this phenomenon is social conformity, which aims to achieve more efficient metabolic expenditure and make one's social environment more predictable by learning what others expect in a given social context. According to this theoretical framework, increased conformity to others in a higher social hierarchy under social observation can be viewed as a more efficient neural strategy for minimizing metabolic costs and uncertainty. In this study, we found no additional neural activity at the time of the observation cue, indicating a public versus private condition when the subject was paired with a superior partner. This finding suggests that conformity to the social hierarchy may be engaged without additional metabolic costs under social observation, probably because the value of conformity to the social hierarchy has already been computed by the vmPFC (and the VS) as well as the rmPFC, even before the cues of public or private conditions. In the private vs public condition, however, we found additional activity in the dmPFC (and the TPJ), which may be responsible for overriding the value of the default decision (i.e., conformity) because the social pressure to conform to a superior partner is now lifted. The motive to maintain consistency of opinion, which could be an alternative way of promoting self-esteem and social position, wins the competition and initiates a value computation for a choice against conformity at the expense of extra metabolic costs.

### Summary and conclusion

In conclusion, this study demonstrated that distinct subregions of the mPFC are differentially involved in SC to social hierarchy under social observation, and these findings seem to be consistent with the recent mPFC model for social valuation where the ventral-to-dorsal gradient of the mPFC subregions are hierarchically structured such that it exerts increasing allostatic control over internalized social responses by adding more information from the external environment (Kim, 2020). We believe these findings provide the first empirical and neural evidence for the novel theoretical framework that SC in the social hierarchy can be best understood as a means of minimizing the metabolic cost of the body (Constant et al., 2019; Theriault et al., 2021), which can contribute to building a novel framework for understanding the complex motivations underlying strategic social conformity. Given that the behavioral and neural processing of social hierarchy can be influenced by cultural differences (Freeman et al., 2009; Chiao, 2010; Shin et al., 2019), it is essential for future research to investigate whether the tendency of strategic conformity in social hierarchical context can be replicated in other cultural contexts such as European and Western cultures.
